# Innovative modified T-shape oncoplastic technique for early-stage breast cancer: multicenter retrospective study

**DOI:** 10.3389/fonc.2024.1367477

**Published:** 2024-06-13

**Authors:** Wenjie Shi, Keqing Li, Wanwan Wang, Xuefeng Shi, Zhongyi Li, Luz Angela Torres-de La Roche, Kai Xu, Rui Zhuo

**Affiliations:** ^1^ Molecular and Experimental Surgery, Faculty of Medicine and University Hospital Magdeburg, Department of General-, Visceral-, Vascular- and Transplant- Surgery, University of Magdeburg, Magdeburg, Germany; ^2^ University Hospital for Gynecology, Pius-Hospital, University Medicine Oldenburg, Oldenburg, Germany; ^3^ Department of Breast and Thyroid Surgery, Xuzhou No.1 People’s Hospital, Xuzhou, Jiangsu, China; ^4^ EUSOMA Certificate Breast Cancer Center (No.1037/00), Guilin TCM Hospital of China, Guilin, China; ^5^ Department of General, Visceral, and Transplant Surgery, Ludwig-Maximilians-University Munich, Munich, Germany

**Keywords:** minimally invasive surgery, oncoplastic surgery, breast-conserving surgery, aesthetic, breast cancer

## Abstract

Inadequate tissue volume at the lower pole of the breast following tumor excision can compromise aesthetic outcomes when employing the conventional inverted-T reconstruction technique. With the aim of reducing postoperative deformities, we have refined this technique. A total of 104 patients underwent the T technique, while 32 underwent the modified T technique and 72 underwent the traditional T technique. In this study, we present the surgical outcomes of the modified T technique group and compare both surgical and oncological outcomes with those of the traditional T technique group. In the modified T technique group, the average tumor size was 23.34 mm, and the mean operation duration was 107.75 min, which was significantly shorter than that of the traditional T technique (*p* = 0.039). Additionally, the average blood loss was 95.93 mL, which was significantly lower than that of the traditional T technique (*p* = 0.011). Although complication rates did not differ significantly between the two groups (*p* = 0.839), the modified T technique yielded superior aesthetic outcomes compared to the traditional T technique (*p* = 0.019). Survival analysis indicated no significant difference in 5-year recurrence-free survival between the two groups, both before and after propensity score matching (*p* = 0.381 vs. *p* = 0.277). As part of our series of oncoplastic techniques for the lower breast quadrant, the modified inverted-T technique utilizes a cost-effective flap to address lower pole defects, mitigating deformities and restoring the breast’s natural shape.

## 1 Introduction

Breast cancer ranks as the most prevalent malignancy among women. Surgical intervention substantially enhances patient prognosis, particularly in early-stage tumors (1, 2). Previously, owing to limited comprehension of the disease and limitations in adjuvant therapy strategies, patients often selected mastectomy rather than breast-conserving surgery (BCS). However, following mastectomy, concerns extend beyond disease progression to psychological distress, encompassing feelings of inadequacy, anxiety, and even depression stemming from breast loss (3, 4). As patient education levels increase, along with growing evidence that supports comparable outcomes between BCS with adjuvant radiotherapy and mastectomy, there has been a shift in patient perspectives, leading to increased acceptance of breast-conserving treatments (5–7).

BCS techniques have limited indications, proving more favorable for patients with less than 20% of the excised glandular volume. However, when the excised glandular volume ranges from 20% to 50%, BCS fails to yield significant aesthetic benefits due to the sizable local defect resulting from tumor excision, posing challenges for repair (8). Additionally, BCS may not consistently deliver satisfactory aesthetic outcomes for all patients, particularly those with small- to medium-volume breasts (9–12). Such breasts possess limited glandular tissue, rendering even excised tissue percentages below 20% irreparable, thus precluding BCS as a viable option. To overcome these challenges, researchers have developed oncoplastic (OPS) techniques, broadening BCS indications while ensuring tumor safety and substantially enhancing post-surgical breast aesthetics (13, 14). Despite the clinical efficacy of OPS techniques, new challenges arise. Empirical evidence suggests that aesthetic prognosis is influenced by tumor site, particularly in OPS applications. Notably, when tumors reside in the lower quadrant, employing the classical inverted-T technique may precipitate bird beak deformities exacerbated by gland and skin contraction, particularly following adjuvant radiotherapy (8, 15). Consequently, the development of novel OPS techniques to augment natural aesthetic outcomes in patients’ breasts becomes necessary.

Based on previous studies, we have developed a series of OPS atlases tailored for breast cancer cases involving tumors in the lower quadrant. These atlases encompass the Zhuo technique, devised to address glandular deficiencies in the inner lower quadrant. This approach enables patients to rectify defects by utilizing adjacent tissue flaps, yielding satisfactory aesthetic breast contours. Additionally, the folded flap technique, designed for the outer lower quadrant of small- to medium-volume breasts, entails folding a flap from the upper abdomen into the defect area via a concealed incision in the inframammary fold (16, 17). This technique markedly ameliorates issues stemming from insufficient self or surrounding tissue glands in small- to medium-volume breasts. In this article, we introduce a modified inverted-T technique tailored for tumors in the lower quadrant of the breast. As an integral component of our lower quadrant atlases, the inverted-T technique represents a refinement of the traditional approach. It involves the use of a tissue flap from the upper abdomen and residual healthy skin above the inferior crease to fill the defect area. This augmentation enhances the abundance of the inferior breast while alleviating tension on retracted breast skin, thereby averting bird beak deformities and restoring the breast’s natural shape.

## 2 Materials and methods

### 2.1 Data collection

**Figure 1:**
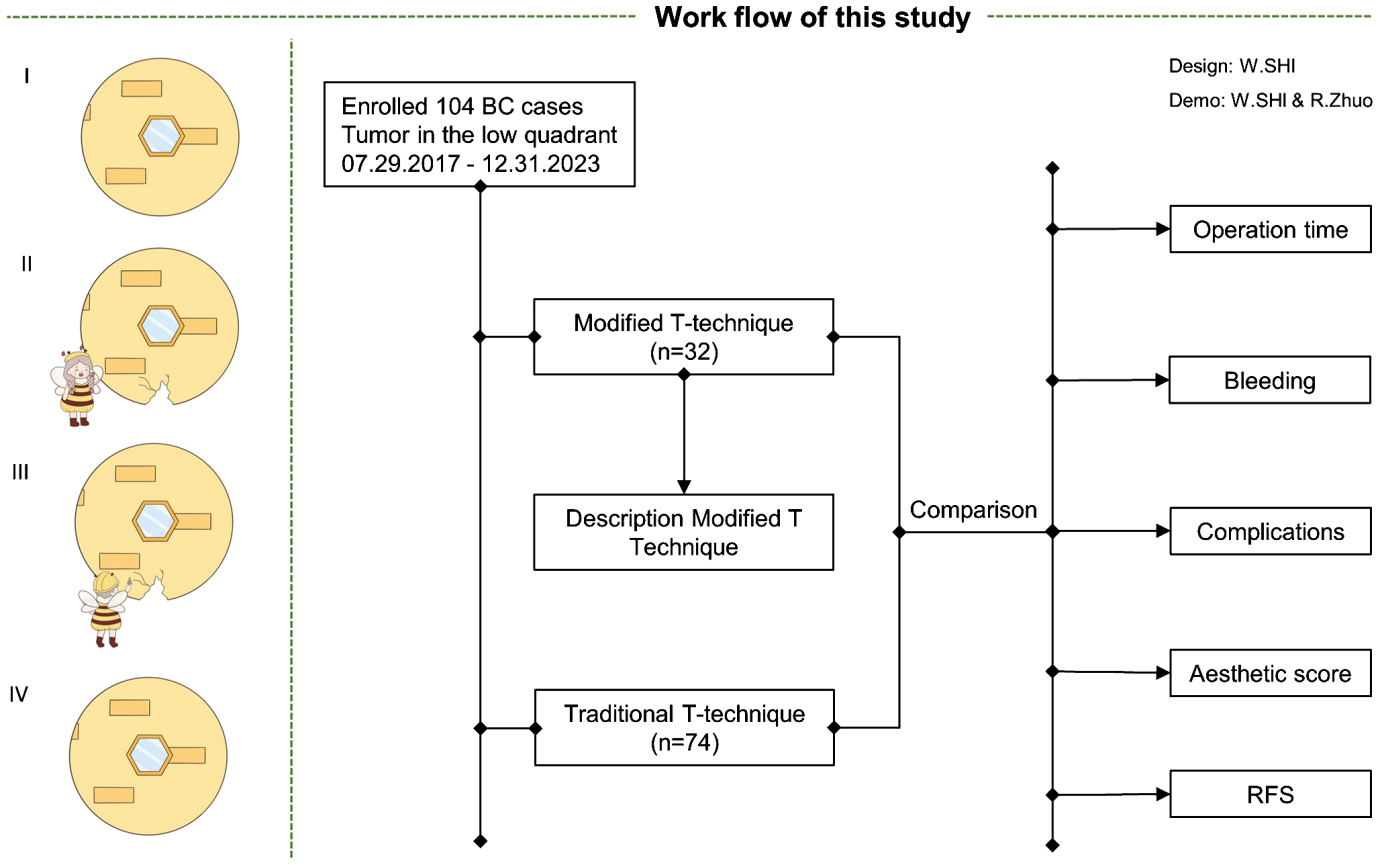
Workflow of our study.

Patient data were obtained from the EUSOMA Certificate Breast Cancer Center (no. 1037/00) and the Department of Breast and Thyroid Surgery, Xuzhou No. 1 People’s Hospital. Enrolled patients provided informed consent, and this study received approval from the Ethics Committee of the EUSOMA Certificate Breast Cancer Center (no. 1037/00) (GTCMH-2023–61; January 2023). The workflow of our study is illustrated in **Figure 1**.

### 2.2 Surgical approach

**Figure 2:**
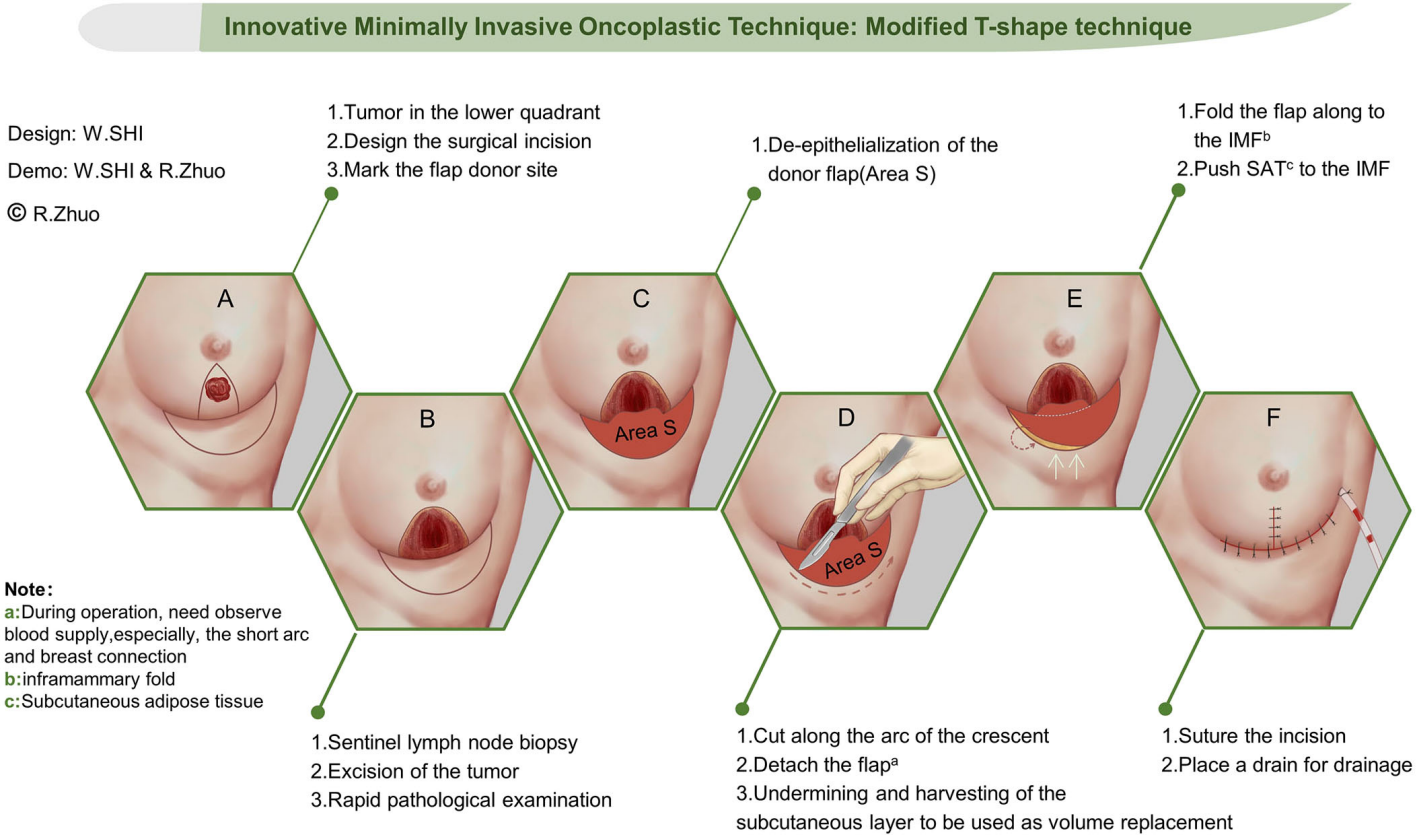
Step-by-step schematic diagram for the modified T-shape technique. Surgery approach design **(A)**, tumor removal **(B)**, de-epithelialization of the donor flap **(C)**, detach the flap **(D)**, repair the defect **(E)**, and closing incision **(F)**. White arrows means up put SAT to the IMF.

The patient is placed in a supine position, and utilizing bedside ultrasound, we identify the mass and delineate a circular area on the skin. Subsequently, arcs are drawn on either side of the circular mark, ensuring that the arc above the mass intersects directly beneath the nipple, while the arcs below the mass intersect on both sides of the folds beneath the affected breast. Our strategy involves utilizing adipose tissue flap from the upper abdominal wall to fill the defect area, necessitating precise determination of flap size. The procedure is performed as follows: the patient stands, and with the inframammary fold as a guide, we push the skin on the upper abdomen to cover the intersection point of the arc above the tumor. Subsequently, we demarcate the crescent-shaped flap area along the arc formed by pushing the skin (**Figure 2A**).

### 2.3 Sentinel lymph node biopsy and tumor resection

Sentinel lymph node biopsy stands as a pivotal stage in BCS, ensuring the efficacy of tumor removal and guiding the necessity for axillary lymph node clearance. Our institution employs a dual tracer technique, utilizing a combination of nuclear and methylene blue dyes for sentinel lymph node biopsy. While awaiting biopsy results, we proceed with tumor removal. We adopt the bilateral arc approach to expose and excise the tumor, defining the resection area as the region surrounding the tumor devoid of ink markings. Subsequently, we mark the specimen on all six sides and send it for rapid freezing, awaiting results. Following confirmation of negative results from both the rapid frozen section and sentinel biopsy, we proceed to the subsequent stage of breast reconstruction (**Figure 2B**).

### 2.4 Flap preparation

As outlined in the previous step, a crescent-shaped flap is marked for the reconstruction of the defect area. In this step, we prepare the flap and utilize it to fill the defect area while restoring the breast’s shape. Initially, we excise the epidermis from the crescent-shaped flap. Notably, we also remove the epidermis from the healthy breast tissue situated above the fold beneath the breast following tumor removal. This flap plays a pivotal role in reconstructing the breast’s shape (**Figures 2C, D**).

### 2.5 Defect repair

Once the flap is prepared, we utilize it to fill the residual cavity. Initially, we empty the retromammary space, followed by folding the crescent-shaped flap along the inframammary fold and toward the posterior gap. This folded flap ensures sufficient glandular tissue to fill the defect area. Additionally, we suture the flap to the preserved healthy breast tissue beneath the breast, facilitating reconstruction of the lower pole of the breast (**Figure 2E**).

### 2.6 Refining the natural shape of breasts

In this stage, reshaping the natural contour of the breast is imperative, with a critical focus on adjusting the lower pole fullness. Initially, we attempt to push the glandular tissue surrounding the residual cavity, suturing it into place. Subsequently, we adjust the breast’s fullness and incision tension while folding the flap. Once a satisfactory breast shape is achieved, we suture the surgical incision in the lower quadrant of the breast. Following this, we free the subcutaneous adipose tissue and push it to the inframammary fold, securing it in place. This procedure effectively conceals the incision. With this, the operation is concluded (**Figure 2F**).

### 2.7 Surgical complications, aesthetic outcomes, and follow-up

Bleeding, infection, subcutaneous fluid accumulation, and flap necrosis represent common complications following BCS, all documented in our center’s database. These complications are promptly managed to ensure timely patient-assisted treatment. Blood loss is calculated based on the volume of fluid in the suction bottles and the saturation of the gauze. It is worth noting that the volume of fluid in the suction bottles includes the amount of irrigation fluid.

The initial assessment of breast aesthetics primarily relies on Paris’s five-point scale (**Supplementary Table 1**), evaluated by the surgeon and assistant during surgery. This scale assesses various criteria including the shape of the affected breast, symmetry between both breasts, and the position of both nipples. A higher score indicates a superior aesthetic outcome for the breast. Subsequently, a second aesthetic evaluation is conducted 1 year postoperatively, jointly performed by the patient and surgeon. In addition to the routine evaluation criteria, particular emphasis is placed on patient satisfaction during this assessment.

## 3 Results

### 3.1 Clinical practice

**Figure 3:**
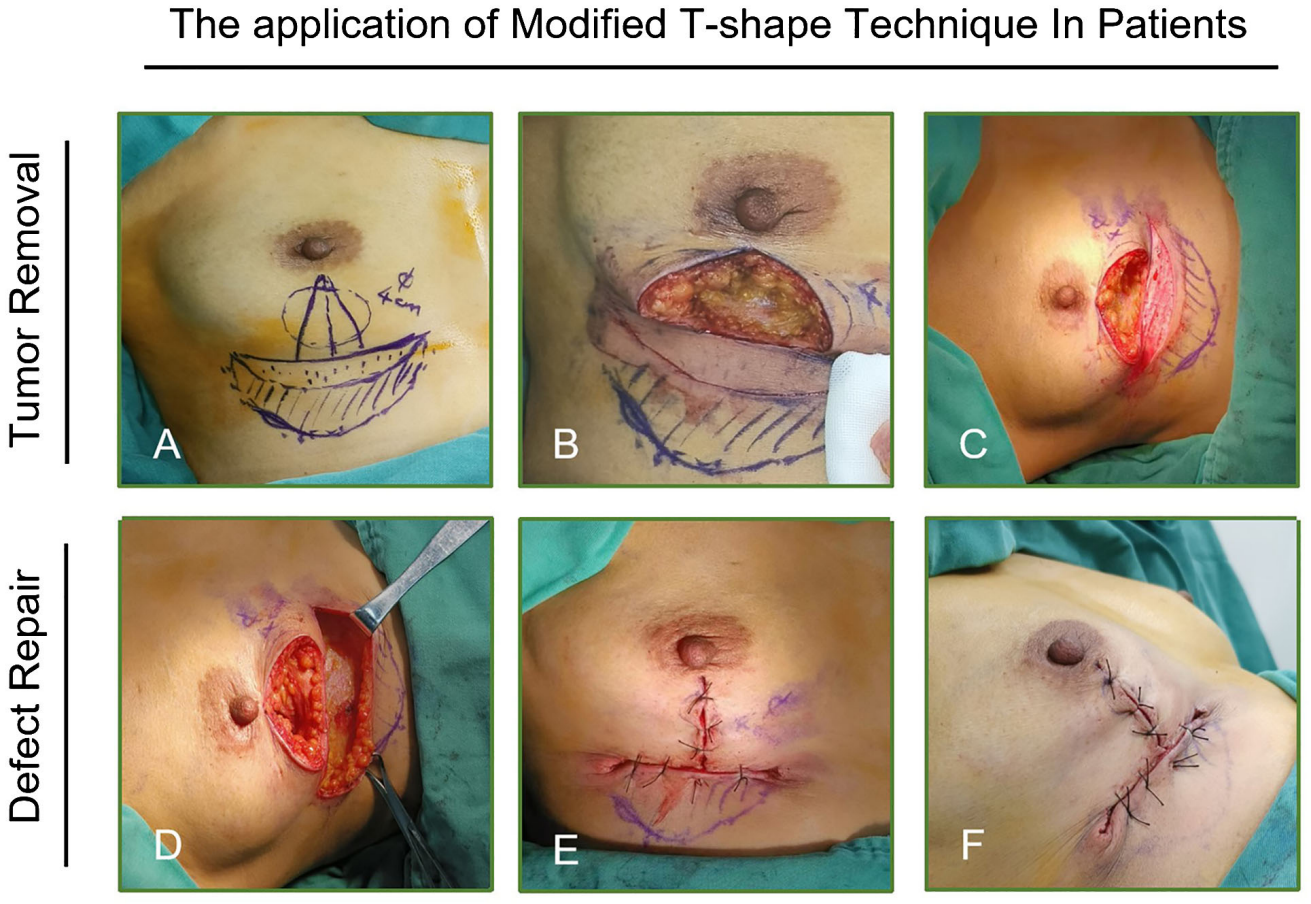
Flap preparation of the modified T technique in clinical practice. **(A)** Surgical approach design, **(B)** tumor resection, **(C)** flap de-epithelialization, **(D–F)** release of flap’s subcutaneous adipose tissue. (The undermining and harvesting of the subcutaneous layer to be used as volume replacement.).

The surgical plan is developed by the breast surgeon, the plastic surgeon, and the patient. The surgery is performed by two breast surgeons who are certified in plastic surgery and one assistant. **Figure 3** depicts the comprehensive operational steps of this technique in real-world settings. In particular, **Figures 3A–C** illustrate tumor localization, surgical incision design, and tumor resection, respectively. **Figures 3D–F** present the defect repair and breast reshaping procedure.

### 3.2 Baseline information

Between 29 July 2017 and 31 December 2023, a total of 104 patients were enrolled in this study. Among them, 32 patients underwent the modified T-shape technique, while 72 patients underwent the traditional T technique. The baseline information for both groups is listed in **Table 1**.

**Table 1:** Baseline characteristics of enrolled patients who underwent the modified T-shape technique.

Characteristics	Overall (*n* = 104)		p
	Modified T technique *n* = 32	Traditional T technique *n* = 72	
**Age (years)**	47.28 ± 10.70	50.60 ± 9.81	0.12
**Tumor size (mm)**	23.34 ± 7.49	20.18 ± 6.46	**0.03**
**BMI^a^ (kg/m^2^)**			1.00
Normal	23 (71.9)	52 (72.2)	
Obesity	9 (28.1)	20 (27.8)	
**Subtype**			0.16
Luminal A	19 (59.4)	26 (36.1)	
Luminal B	8 (25.0)	32 (44.4)	
HER2 positive	3 (9.4)	8 (11.1)	
TNBC	2 (6.2)	6 (8.3)	
**Quadrant**			0.64
Low half	8 (25.0)	13 (18.1)	
Low inner	14 (43.8)	31 (43.1)	
Low outer	10 (31.2)	28 (38.9)	
**Histology**			0.89
IDC^b^	28 (87.5)	66 (91.7)	
ILC^c^	1 (3.1)	2 (2.8)	
MAC^d^	1 (3.1)	1 (1.4)	
MCB^e^	2 (6.2)	3 (4.2)	
**CUP**			0.18
A	16 (50.0)	30 (41.7)	
B	13 (40.6)	24 (33.3)	
C	3 (9.4)	18 (25.0)	
**Diabetes history**			0.30
Yes	0 (0)	5 (6.9)	
No	32 (100)	67 (93.1)	

^a^Body mass index; ^b^Invasive ductal carcinoma; ^c^Invasive lobular carcinoma; ^d^Mucinous adenocarcinoma; ^e^Medullary carcinoma of breast. Bolded values means p value less than 0.05.

For patients undergoing the modified T-shape technique, the average age is 47.28 years (24–70 years). Notably, 11 of these patients were classified as young breast cancer patients, with ages < 45 years.

Among patients undergoing the modified T-shape technique, 9 had a body mass index (BMI) exceeding 25 kg/m^2^, while the remaining 23 had normal BMI (18.5–25 m^2^). The majority of patients presented with A cup breasts, accounting for 50.0% (16 of 32), followed by B cup (40.6%, 13 cases), and C cup (9.4%, 3 cases). The average tumor size was 23.34 mm (10–52 mm), predominantly consisting of invasive ductal carcinoma in 87.5% of cases (28 cases). Additionally, one case each was identified as invasive lobular carcinoma and mucinous adenocarcinoma, with two cases of medullary carcinoma of breast. Molecular subtype distribution primarily comprised luminal A and B subtypes, accounting for 59.4% and 25.0% of cases, respectively. Additionally, three cases exhibited human epidermal growth factor receptor 2 (HER2)-positive subtypes, while two cases presented with triple-negative breast cancer subtype.

For patients undergoing the traditional T technique, the average age was 50.60 years, with an average tumor size of 20.18 mm. The predominant subtype was luminal B, comprising 44.4% (32 of 72) of cases. In terms of histological type, invasive ductal carcinoma is predominant, representing 91.7% (66 of 72) of cases. Additionally, five patients in this group have a history of diabetes.

### 3.3 Surgical outcomes

**Figure 4:**
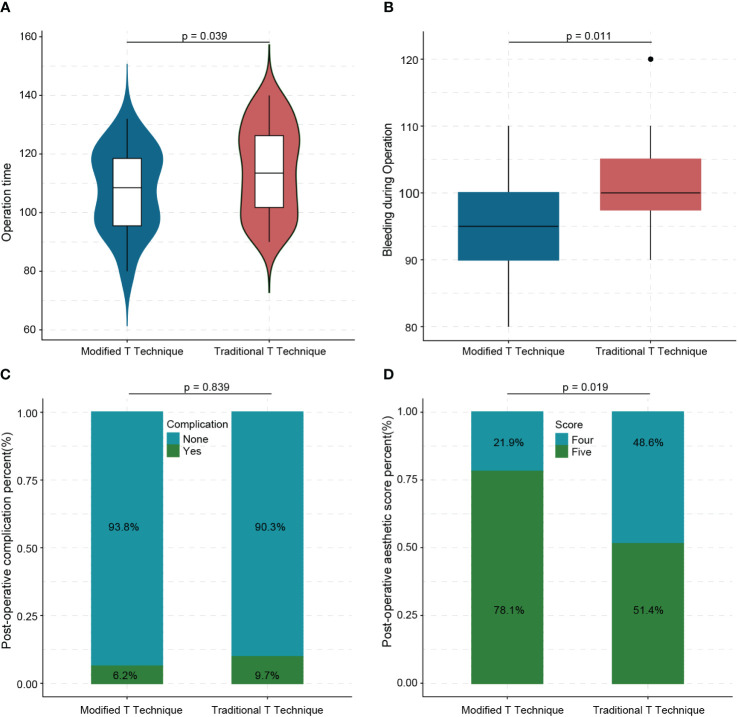
Difference between modified T technique and traditional T technique in operation time **(A)**, bleeding **(B)**, complications **(C)**, and aesthetic outcomes **(D)**.

In patients undergoing the modified T-shape technique, the average operation time was 107.75 min (80–132 min), which was significantly shorter than that of patients undergoing the traditional T technique (114.58 min) (*p* = 0.039) (**Figure 4A**). Furthermore, the average blood loss was 95.93 mL (80–110 mL), significantly lower than that of patients undergoing the traditional T technique (100.97 mL) (*p* = 0.011) (**Figure 4B**). Intraoperative rapid frozen section analysis revealed negative margins without ink staining in all patients, preventing the need for second resection. In the modified T-shape technique group, one patient experienced postoperative bleeding, while another patient exhibited local skin redness and swelling. Conversely, seven patients in the traditional T technique group experienced postoperative complications (*p* = 0.839) (**Figure 4C**). Postoperative bleeding was managed effectively with compression bandage application, resolving the symptoms. Patients presenting with redness and swelling received antibiotic prophylaxis, effectively controlling the symptoms. Notably, there were no reports of fat liquefaction or flap necrosis.

### 3.4 Aesthetic outcomes

**Figure 5:**
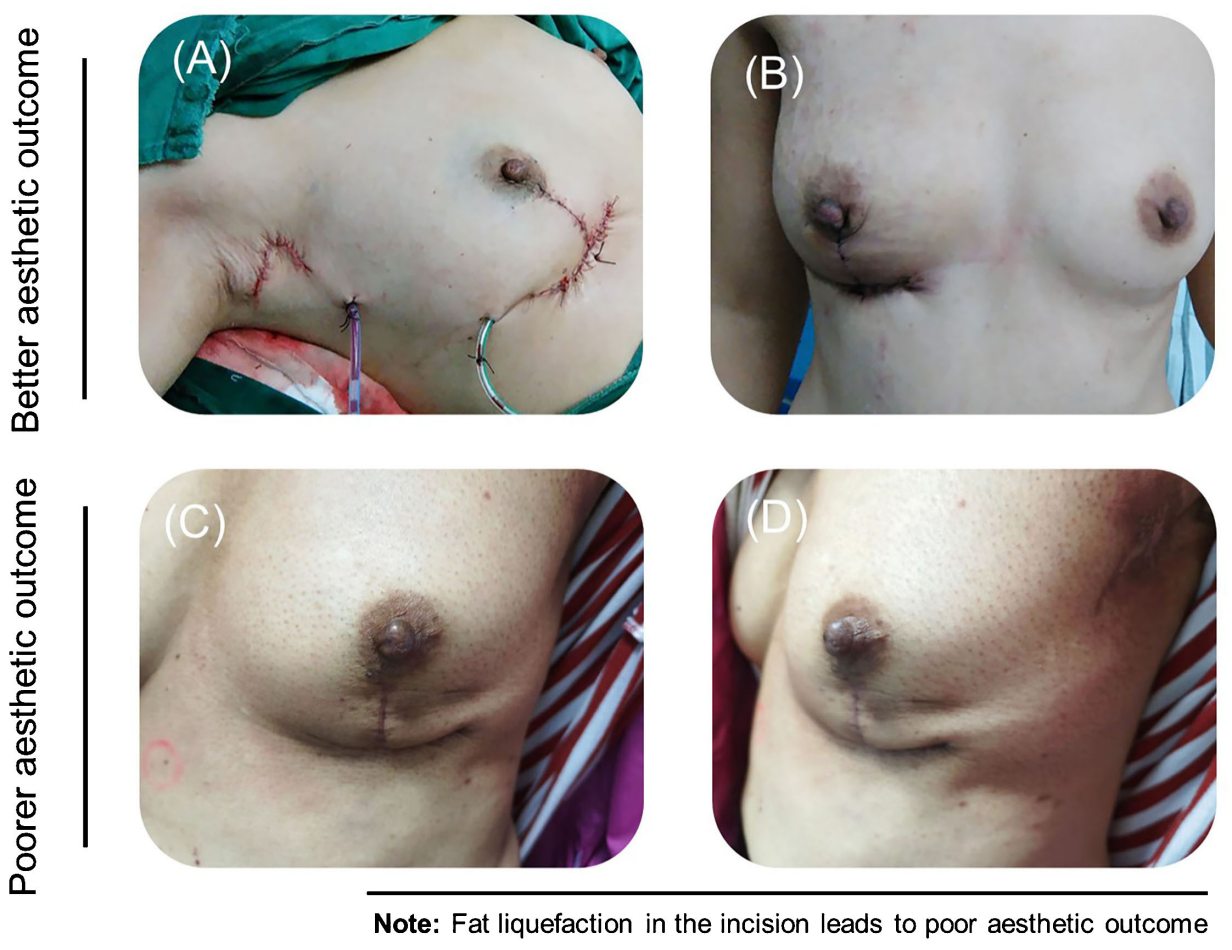
Aesthetic outcome of patients who received the modified T-shape technique, including immediate postoperative **(A)** and aspect of 3 months after surgery **(B)**. The deformity of the dog ears in another patient is shown in the front **(C)** and from the side **(D)**.

Immediate postoperative aesthetic evaluation revealed that the reconstructed breasts exhibited a natural shape, rounded inferior fullness, symmetrical bilateral nipples, and no displacement of the nipple–areola complex. After 1 year, the average aesthetic evaluation score among patients in the modified T-shape technique group was 4.78 (4–5) points. This score signifies a highly natural breast shape. Moreover, it was significantly higher than the average score of patients who received the traditional T technique (*p* = 0.019) (**Figure 4D**). The aesthetic outcome of patients who received the modified T-shape technique, including immediate postoperative (B) and aspect of 3 months after surgery, is shown in **Figures 5A, B**. **Figures 5C, D** display a poor outcome of patients with a dog ears deformity from the front and side.

### 3.5 Long-term follow up

**Figure 6:**
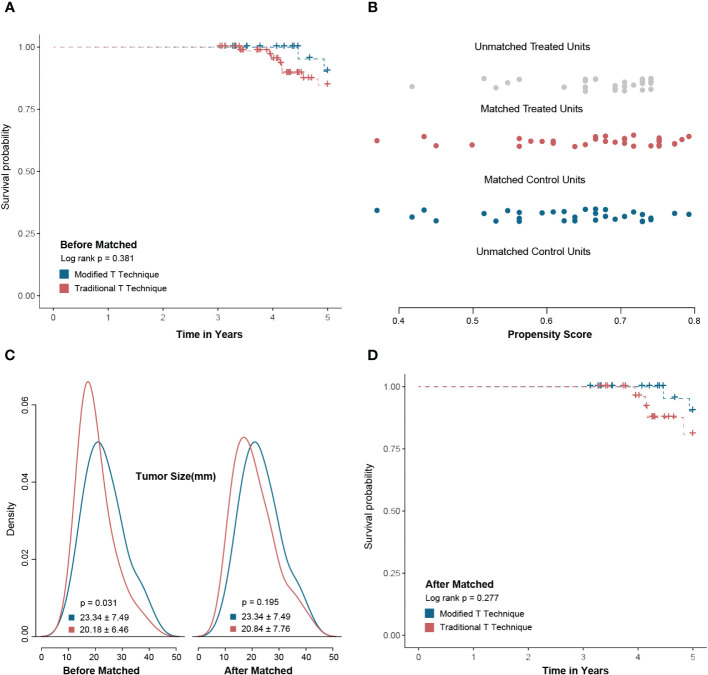
Survival analysis between the modified T technique and traditional T technique. There was no significant 5-year recurrence-free survival between modified T technique and traditional T technique groups before propensity-score matching (PSM) **(A)**. After PSM by tumor size, the baseline factors were balanced between two groups **(B, C)**. After PSM, there was no significant difference in 5-year recurrence-free survival between the modified T technique and traditional T technique groups **(D)**.

The median follow-up time for patients after surgery was 61.61 (37.58–80.25) months. Registry data revealed that one patient with luminal A subtype and a high-risk score of 21-gene experienced local recurrence, while another patient in the modified T-shape technique group declined chemotherapy, resulting in tumor recurrence. Survival analysis indicated that the 5-year recurrence-free survival did not significantly differ between the two groups, both before and after propensity score matching (*p* = 0.381 and *p* = 0.277, respectively) (**Figures 6A–D**).

## 4 Discussion

The traditional inverted-T technique often proves challenging for repairing tumors in the lower quadrant of the breast and frequently results in bird beak deformity. In clinical practice, researchers have identified insufficient lower pole tissue volume and excessive skin tension as primary contributors to this deformity (18, 19). In response, we have developed a modified T-shape technique, which effectively circumvents the occurrence of breast deformities post-tumor removal. This technique represents a significant advancement in enhancing the aesthetic outcomes for patients undergoing breast surgery.

Similar to the surgical approach of the traditional inverted-T technique, the modified T-shape technique also operates around the inframammary fold. However, a key distinction lies in the preservation of the inframammary fold’s original structure in our technique, serving solely as a reference line to aid in flap folding. This preservation reduces the risk of complications arising from inframammary fold damage. Conversely, the inframammary fold is incised in the traditional inverted-T technique for better surgical exposure (20). Additionally, while the traditional inverted-T technique relies on surrounding glandular tissue to fill the residual cavity post-tumor resection, this approach may encounter challenges when the breast volume is insufficient, potentially leading to deformities (8). Conversely, the modified T technique offers a solution to gland insufficiency by utilizing an upper abdominal free flap. This flap is folded into the residual cavity alongside the remaining healthy tissue, effectively augmenting the lower pole of the breasts and mitigating the risk of post-surgical deformity. The detailed difference is presented in **Supplementary Table 2**.

In addition to the inverted-T technique, the classic V-shape and J-shape techniques are also commonly utilized in the treatment of tumors in the lower quadrant of the breast (13, 21). The V-shape technique achieves tumor resection through segmental resection, presenting as a straightforward and easily mastered approach by clinicians. However, in our clinical experience, patients predominantly present with small- to medium-volume breasts. Consequently, preoperative evaluation often reveals gland insufficiency when considering a simple V-shaped technique. Thus, compared to this approach, the modified inverted-T technique appears more suitable for such patients. Our technique utilizes surrounding breast flap to fill the defect during tumor resection, effectively overcoming the challenge of gland insufficiency. Regarding the J-shape incision, its elongated nature and increased risk of breast deformity hinder its widespread adoption. In contrast, our incision design consists of two parts, with the lower crease incision concealed due to breast gravity. The exposed vertical incision is significantly shorter than the J-shaped incision, ensuring tumor safety while yielding a more natural breast shape. Postoperative complications can impede incision healing and delay the application of adjuvant therapy (13). In our postoperative follow-up, three patients experienced varying degrees of complications, which were effectively managed with corresponding treatment, thus averting delays in adjuvant therapy initiation. Notably, one patient undergoing this surgery experienced local recurrence of the luminal A subtype. High-risk results from 21-gene detection may have contributed to this recurrence, despite indications suggesting potential benefits from chemotherapy.

In addition, researchers have reported on the use of the AICAP flap for breast reconstruction. Unlike our technique, this technology can determine the position of the flap based on the course of different perforators, making it very favorable for the lower outer quadrant of the breast. Moreover, this technique can also provide enough glandular tissue to repair defects. However, this technique requires the surgeon to possess a certain level of anatomical skills, which indirectly increases the operational difficulty of the surgery compared to our easy-to-perform technology (22).

Currently, the findings from retrospective analysis indicate that our technique is conducive to tumor resection in the lower quadrant of the breast. However, further validation from multiple breast centers is warranted to ascertain its clinical value. Nevertheless, it is crucial to acknowledge the limitations of this surgical approach.

We do not recommend this technique for patients with low BMI because obtaining an upper abdominal flap is more difficult, and the flap does not provide sufficient glandular tissue for the defect. Additionally, if the tumor is situated close to the inframammary fold, this technique may not be applicable. This is because tumor removal may compromise the integrity of the inframammary fold, potentially leading to flap necrosis due to inadequate blood supply.

## 5 Conclusions

Similar to other OPS techniques developed by our group, the surgical and oncological outcomes of this case series suggest that the modified T technique is feasible for patients with small- to medium-sized breasts and tumors located in the mid-line of the lower pole of the breast. This technique offers a favorable and adaptable flap to fill the residual cavity post-tumor resection, significantly augmenting the lower pole of the breast and mitigating the risk of breast deformity while preserving the natural breast shape. Furthermore, we hypothesize that in cases of large breasts, the final horizontal scar will be concealed once the patient assumes the supine position. However, it is imperative to acknowledge that the clinical utility of this modified technique requires validation through comparative studies.

## Data availability statement

The original contributions presented in the study are included in the article/**Supplementary Material**. Further inquiries can be directed to the corresponding authors.

## Ethics statement

The studies involving humans were approved by the Ethics Committee of EUSOMA Certificate Breast Cancer Center (No.1037/00). (GTCMH-2023-61; January 2023). The studies were conducted in accordance with the local legislation and institutional requirements. The participants provided their written informed consent to participate in this study. Written informed consent was obtained from the individual(s) for the publication of any potentially identifiable images or data included in this article.

## Author contributions

WS: Conceptualization, Data curation, Formal analysis, Visualization, Writing – original draft, Writing – review & editing. KL: Conceptualization, Investigation, Methodology, Project administration, Resources, Software, Writing – original draft. WW: Conceptualization, Formal analysis, Methodology, Project administration, Resources, Writing – review & editing. XS: Investigation, Methodology, Project administration, Resources, Writing – review & editing. ZL: Conceptualization, Data curation, Formal analysis, Methodology, Resources, Software, Visualization, Writing – review & editing. TL-A: Formal analysis, Investigation, Methodology, Visualization, Writing – review & editing. KX: Conceptualization, Data curation, Project administration, Resources, Supervision, Writing – original draft, Writing – review & editing. RZ: Conceptualization, Formal analysis, Funding acquisition, Investigation, Methodology, Project administration, Resources, Supervision, Validation, Writing – review & editing.

## Supplementary material

Supplementary Figure 1 Incision design of the modified T-shape technique.
