# Supplementary figures and images for: Innovative modified T-shape oncoplastic technique for early-stage breast cancer: multicenter retrospective study
Source: Front Oncol. 2024 Jun 13;14:1367477. doi: 10.3389/fonc.2024.1367477 (PMC11208303; doi:10.3389/fonc.2024.1367477)

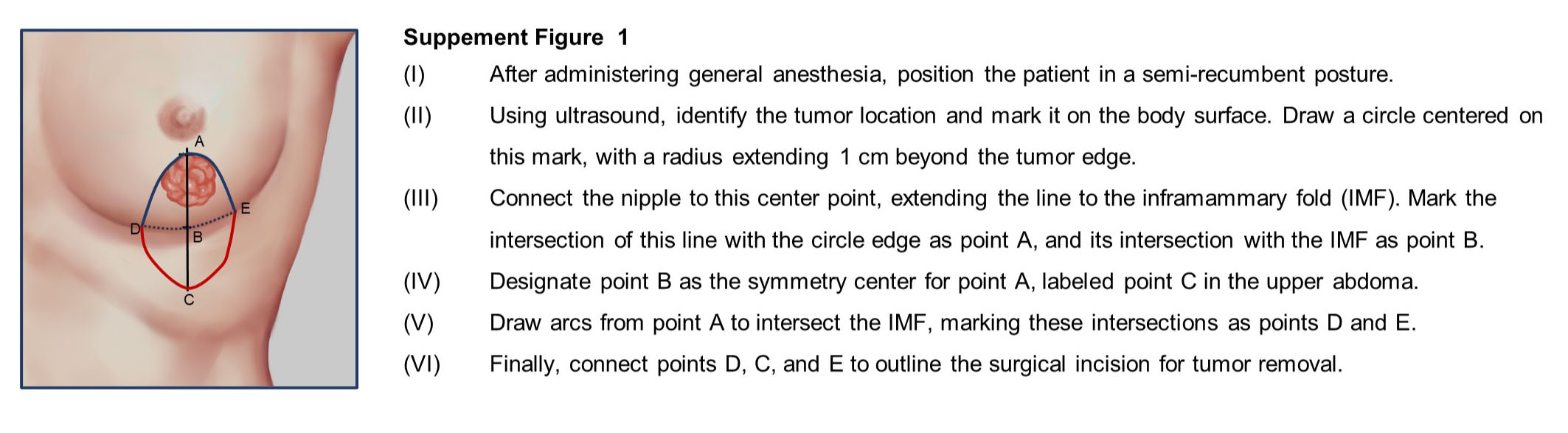

Supplement: Supplementary Figure 1 — Incision design of the modified T-shape technique. [file Image_1.jpeg]
