# Supplementary material for: Innovative modified T-shape oncoplastic technique for early-stage breast cancer: multicenter retrospective study
Source: Front Oncol. 2024 Jun 13;14:1367477. doi: 10.3389/fonc.2024.1367477 (PMC11208303; doi:10.3389/fonc.2024.1367477)
Supplement: Supplementary file 2 [file Table_1.docx]

**Supplement Table 1: The Paris five-point scale to rate cosmetic outcomes**

| **Score** | **Overall aesthetic results** | **Breast forms** | **NAC positions** | **Action required** |
| --- | --- | --- | --- | --- |
| 5 | Excellent | No detectable asymmetry | Perfect position | No further action |
| 4 | Good | Perfect breast form with minimal asymmetry. | Good position | No further action |
| 3 | Fair | Minimal retraction in the operated quadrant / asymmetry of volume. | Minimal deviation of NAC toward the operated quadrant | ASCT type 1 requiring contralateral symmetrization |
| 2 | Poor | Deformation of' operated breast. | Deviation of NAC | ASCT type 2 requiring ipsilateral reoperation |
| 1 | Bad | Major deformation of operated breast. | Distortion of NAC | ASCT type 3 mastectomy |
