# Supplementary material for: Innovative modified T-shape oncoplastic technique for early-stage breast cancer: multicenter retrospective study
Source: Front Oncol. 2024 Jun 13;14:1367477. doi: 10.3389/fonc.2024.1367477 (PMC11208303; doi:10.3389/fonc.2024.1367477)
Supplement: Supplementary file 3 [file Table_2.docx]

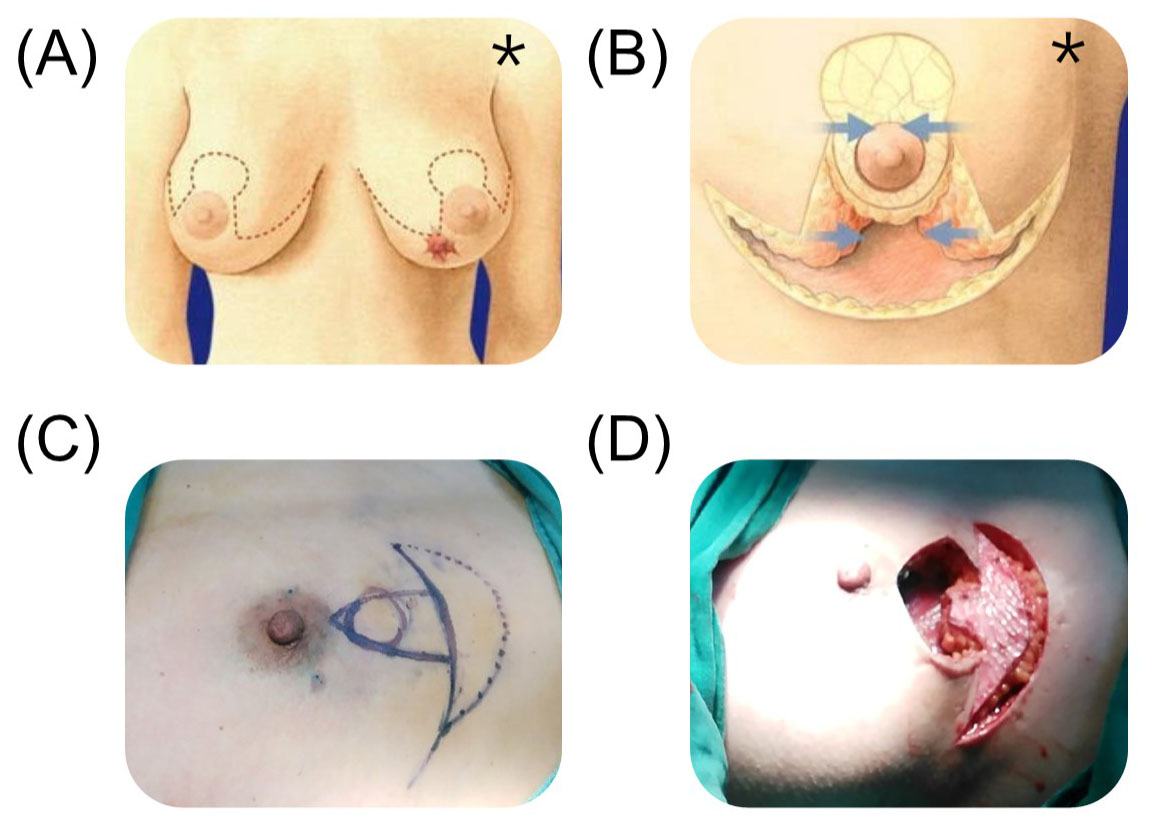


**^*^Source from:** Clough, Krishna B., et al. "Improving breast cancer surgery: a classification and quadrant per quadrant atlas for oncoplastic surgery." Annals of surgical oncology 17 (2010): 1375-1391.(**Figure A & B**)

**Supplement Table 2:** The difference between Traditional T and Modified T technique

| Figures | A&B | C&D |
| --- | --- | --- |
| Techniques | Traditional T technique | Modified T technique |
| Design of incision | Breasts surface | Upper abdomen |
| Glandular source | Breast | Upper abdomen flap |
| Difficulty of surgery | Middle | Low |
